# Phloroglucinol inhibited glycation *via* entrapping carbonyl intermediates

**DOI:** 10.1371/journal.pone.0307708

**Published:** 2024-07-25

**Authors:** Hammad Ahmed, Talha Bin Fayyaz, Najeeb Khatian, Shumaila Usman, Uzair Nisar, Mohammad Abid, Syed Abid Ali, Ghulam Abbas

**Affiliations:** 1 Department of Pharmacology, Faculty of Pharmacy, Ziauddin University, Karachi, Pakistan; 2 Department of Molecular Medicine, Ziauddin University, Karachi, Pakistan; 3 H.E.J. Research Institute of Chemistry, International Center for Chemical & Biological Sciences, University of Karachi, Karachi, Pakistan; Integral University, INDIA

## Abstract

Advanced glycation end products (AGEs) play an important role in the pathogenesis of age-linked disorders and diabetes mellitus. The aim of this study was to assess the repurposing potential of Phloroglucinol (PHL the antispasmodic drug), as an anti-glycation agent using Fructose-BSA model. The ability of PHL to inhibit AGE formation was evaluated using AGEs formation (Intrinsic fluorescence), fructosamine adduct (NBT) and free lysine availability (TNBSA) assays. The BSA protein conformation was assessed through Thioflavin-T, Congo-Red and Circular Dichroism assays. The lysine blockade and carbonyl entrapment were explored as possible mode of action. Our data showed that PHL significantly decreased the formation of AGEs with an IC_50_ value of 0.3mM. The fructosamine adducts and free lysine load was found to be reduced. Additionally, the BSA conformation was preserved by PHL. Mechanistic assays did not reveal involvement of lysine blockade as underlying reason for reduction in AGEs load. This was also supported by computational data whereby PHL failed to engage any catalytic residue involved in early fructose-BSA interaction. However, it was found to entrap the carbonyl moieties. In conclusion, the PHL demonstrated anti-glycation potential, which can be attributed to its ability to entrap carbonyl intermediates. Hence, the clinically available antispasmodic drug, presents itself as a promising candidate to be repurposed as anti-glycation agent.

## Introduction

Ageing is defined as the progressive loss bodily functions with time. One of the potential underlying explanation to this is the phenomenon of glycation [1]. The glycation, also termed as Maillard’s reaction, involves cascade of events, which begins with the non-enzymatic interaction of proteins with sugars. This is followed by reactions such as dehydration, condensation, cyclisation and fragmentation, which ultimately leads to the formation of compounds termed as Advanced Glycation End products [2, 3]. The rate of protein glycation is dependent upon various factors such as presence of free amino groups on the N-terminus (such as arginine and lysine). The glycation phenomenon is also facilitated by the presence of histidine residue in close proximity with lysine [4]. Furthermore, the acidic and basic nature of the neighboring groups also affect the rate of glycation *via* altering the pKa of amino groups. They either augment the nucleophilicity or catalyze the Amadori rearrangement, which is considered as the rate limiting step in process of protein glycation [5].

The glycation phenomenon causes the change in conformation of proteins thereby leading aggregation and loss of function [6]. These aggregated proteins are resistant to proteolytic enzymes, and thus accumulates in the body and contribute towards ageing. Moreover, they have been attributed to cognitive impairment [7], diabetic complications [8], renal [9] and hepatic [10] insufficiencies. Additionally, the AGEs also binds to its receptor termed as RAGE (receptor for AGES) and leads to stress specifically termed as glycative stress [11–13].

**Fig 1:**
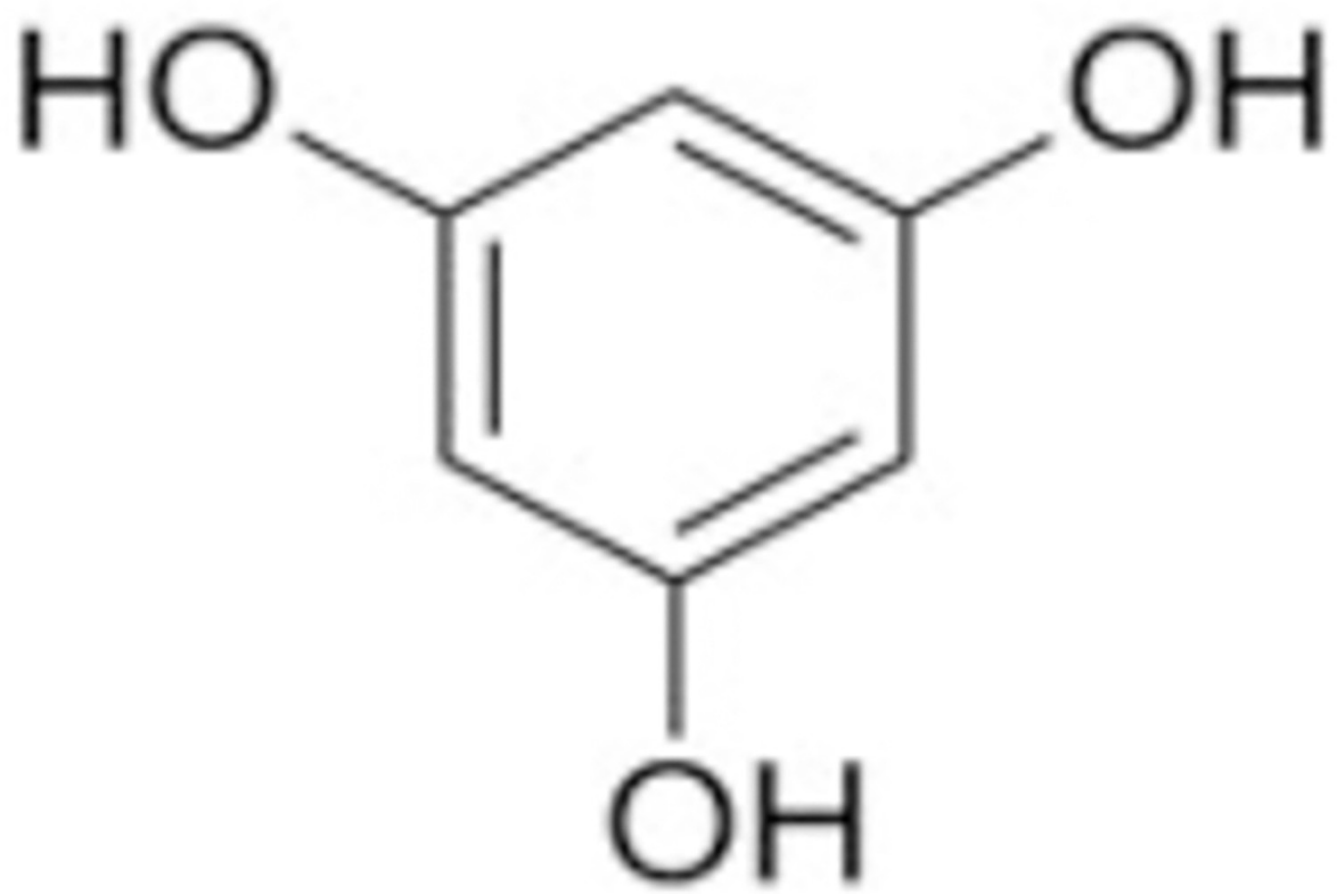
Structure of Phloroglucinol.

Numerous agents have been reported to halt the process of glycation. The foremost popular among these agents is Aminoguanidine, which is a carbonyl entrapper [14]. Unfortunately, none of them could pave their way to bedside due to diverse issues especially adverse effects. Under this scenario, the concept of repurposing offers robust method to introduce the newer molecule(s) for drug discovery program [15]. Phloroglucinol (PHL; benzene-1,3,5-triol, Fig 1) is a type of phloro-tannin, which is commonly found in marine brown algae [16]. It is clinically used for the treatment of abdominal pain [17]. Additionally, it has anti-infective [18] and antioxidant [19] properties.

Keeping the concept of repurposing in mind, the present study was designed to evaluate Phloroglucinol (PHL) for its capacity to be repurposed as anti-glycation agent.

## Materials and methods

### Chemicals

The following chemicals were used in the study: 2-methylquinoxaline, 5-methylquinoxaline and *O*-Phthaldialdehyde (Alfa Aesar, USA). Aminoguanidine and Nα-acetyl-l-lysine (Chem Cruz, USA). Bovine serum albumin (Sigma Aldrich, USA), β-mercapethanol, Congo Red, *O*-Phenyldiamine and Sodium bicarbonate (Sigma Aldrich, USA). 2,4,6-trinitrobenzenesulfonic acid, Methylthiazolyldiphenyl-tetrazolium bromide, D- fructose, Formic acid, Sodium carbonate, Sodium dihydrogen phosphate, Sodium hydrogen phosphate and Phosphate buffer saline tablet (Sigma Aldrich, USA). (Dimethyl sulfoxide (Merck, USA). Ethanol 100% (Serva, Germany). Phloroglucinol (Himont Pharma, Pakistan). Nitro blue tetrazolium and Thioflavin T (Santa Cruz Biotechnology, USA). Sodium azide and Sodium dodecyl sulfate (Kanto chemical, Japan).

### Anti-glycation assay

The assay was performed by preparing the reaction mixtures, which contained nBSA (10 mg/ml in phosphate buffer 0.2 M, pH 7.4) alone or in combination with fructose (200 mM) in the absence or presence of Aminoguanidine (3 mM) or Phloroglucinol (0.5, 1, and 2 mM). These reaction mixtures were heated at 60°C for 24 h followed by measurement of intrinsic fluorescence (excitation and emission at 360 nm and 460nm respectively) using spectrofluorometer (JASCO, Japan) as described earlier [20].

### Fructosamine adduct assay

The test was performed on aforementioned reaction mixtures using NBT dye. The sample containing reaction mixtures and NBT dye (0.25 mM in carbonated buffer of pH 10.35) were incubated for 2 h. Afterwards, the absorbance was measured at 525 nm using spectrophotometer (JASCO, Japan) as described earlier [21].

### TNBSA assay

The stock solution of TNBSA (0.01% w/v) was prepared in sodium bicarbonate buffer (0.1M, pH 8.5). The aforementioned reaction mixtures were mixed with TNBSA solution and incubated for 2 h. After incubation, the absorbance was checked at 335nm using Spectrophotometer (JASCO, Japan) as described earlier [22].

### Thioflavin-T assay

The stock solution of ThT (100 μM) was prepared in phosphate buffer (pH 7.3, 5ml). The reaction mixtures (40 μl) were mixed with ThT stock solution (160 μl) and incubated. Later, the fluorescence was measured (Excitation and emission lambda of 460 and 485 nm, respectively) using Spectrofluorometer (JASCO, Japan) as described earlier [23].

### Congo Red assay

The stock solution of CR dye (12.5 M) was prepared in Tris-HCl buffer (pH 7.3). The CR stock solution (40 μl) was mixed with aforesaid reaction mixtures (160 μl) and allowed to stay at ambient temperature for 20 min. Subsequently, the absorbance was measures at 530 nm using spectrophotometer (JASCO, Japan) as described earlier [24].

### Circular dichroism

The secondary structure of BSA in the reaction mixture was evaluated using JASCO J810 Spectropolarimeter (Japan). Glycated protein samples were scanned at least four times in the UV (250–400 nm) and Far-UV amide regions at room temperature to obtain spectra. The secondary structural modification of protein was estimated through CD spectral analysis using the online Dichro Web server [25].

### Lysine blockade assay

The OPA (o-phthaldialdehyde) assay was used to assess the lysine blockade activity of PHL as reported earlier [26]. The OPA reagent (100 ml) was prepared by mixing OPA (80 mg) in absolute ethanol (2 ml), SDS (5 ml, 20%), β-Mercapethanol (200 μl), and sodium tetraborate buffer (50 ml, pH 10). A protein sample (25 μg, 50 μl liquid BSA) was dissolved in de-ionized water containing PHL and incubated for 15 min to estimate the lysine blockade. The fluorescence was checked immediately (excitation and emission lambda of 360 and 460 nm, respectively) to estimate the available epsilon lysine amino group using spectrofluorometer (JASCO, Japan). For the calibration curve, N-α-acetyl-lysine (dissolved in 0.1% formic acid) was used using different concentrations ranging from 50 100 μM.

### Computational study

The 3D crystal structure of BSA (PBB ID: 4F5S) was obtained from protein data bank. The 2D structure of the ligand i.e. Phloroglucinol was downloaded (PUBCHEM) as SDF file and converted to 3D format (PYMOL 3.11). Later, the target molecular docking studies was performed by using Auto dock vina 1.1.2 [27]. The receptor and ligand were transformed to pdbqt format for docking. The active sites were predicted by Prank web database and the grid was set as x = 100, y = 100 and z = 100 along with the coordinate sizes as x = 4.368, y = 16.991 and z = 106.819, respectively. Once docking was completed, the most stable confirmation of ligand-protein interaction was used for the analysis of docking results with the help of Biovia Discovery tool 2021 client.

### Carbonyl entrapping assay

The phosphate buffer saline (0.1 M, pH 7.4) was used to prepare methyl glyoxal solution (MGO, 0.4 mg/ml). The reaction mixture was prepared by dissolving MGO (100 μl), PBS (870 μl, 0.1 M, pH 7.4) and 5-MQ (50 μl, negative control). The AG (5 mM) was used as a positive control. The PHL capacity to trap carbonyls was evaluated at 0.5, 1, and 2 mM strengths. The test agents (100 μl) were mixed with 900 μl of aforementioned reaction mixture. After vortex, the samples were heated for 24 h at 60°C. Afterwards, OPD (200 μl) was added and re-incubated for 30 min in the dark to finish the derivatization reaction. The amount of 2-MQ found in each sample was used to quantify the MGO residue using HPLC system (Shimadzu Prominence) equipped with auto-sampler (SIL-20 A), pump (LC-20 A), and diode array detector (SPD-M20A). The CBM-20 A linked software (LC solutions) and hardware. For chromatography separation, Hibar® 250, 4–6 Lichrospher® RP C18e (5 μ) column was used preceded by nucleosil 100–5 C18 guard column. A mixture of 1:1 (HPLC grade) methanol and filtered deionized water with 5% glacial acetic acid (HPLC grade) makes up the mobile phase. The samples were filtered (0.22 μm syringe filter) and injected into HPLC. The flow rate was maintained at 1 ml/min for a duration of 20 min. The 2-MQ area under the curve was used to estimate the carbonyl entrapment [28].

### Statistical analysis

The data is presented as mean ± SEM (n = 3). The statistical analysis was done by using one-way ANOVA (IBM-SPSS, USA). The existence of hash (#) or asterisks (*) as single, double or triple represents the different levels of significance (p<0.05, p<0.01 and p<0.001) as compared to respective controls i.e. nBSA and gBSA.

## Results

### AGE inhibition assay

**Fig 2:**
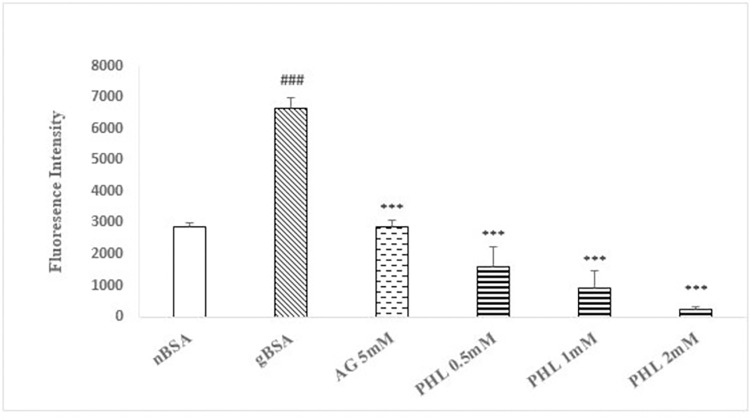
Effect of Phloroglucinol on AGES formation. The figure shows the formation of advance glycation end products (as intrinsic fluorescence) in the presence of AG and PHL. The gBSA group exhibit enhanced fluorescence as compared to nBSA. The AG and PHL has significantly reduced the formation of AGEs in dose dependent manner. The hash (###) represents the significant (p<0.005) difference as compared to nBSA while asterisks [* (p<0.05), ** (p<0.01) and ***(p<0.005)] represents the statistical comparison with the gBSA. All values are expressed as mean ± SEM of intrinsic AGEs fluorescence intensity (n = 3).

The gBSA showed significant (p<0.005) increase (130%) in the intrinsic fluorescence intensity as compared to nBSA (Fig 2). The standard drug AG (5 mM) caused significant reduction (p<0.005, 57%) in the fluorescence intensity as compared to gBSA. The PHL treatment (0.5, 1 and 2 mM) exhibited significant (p<0.005) dose dependent decrease (97%, 68% and 45% respectively) in fluorescence intensity with an IC_50_ value of approximately 3mM.

### Fructosamine adduct assay

**Fig 3:**
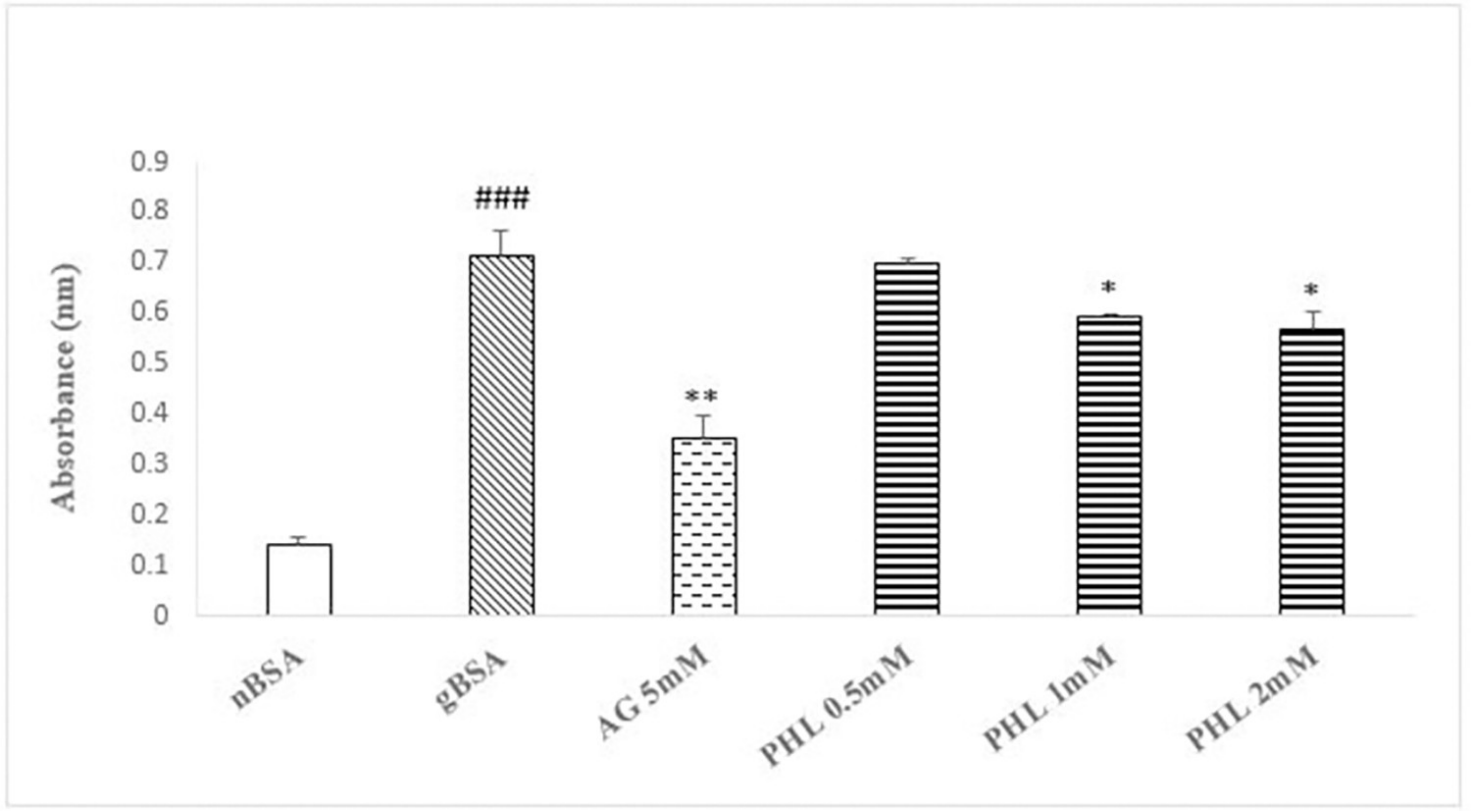
Effect of Phloroglucinol on fructosamine adduct assay. The figure shows the formation of fructosamine adducts as absorbance in the presence of AG and PHL. The gBSA group exhibit enhanced abosrbance as compared to nBSA. The AG and PHL has significantly reduced the formation of adducts in dose dependent manner. The hash (###) represents the significant (p<0.005) difference as compared to nBSA while asterisks [* (p<0.05), ** (p<0.01) and ***(p<0.005)] represents the statistical comparison with the gBSA. All values are expressed as mean ± SEM of absorbance (n = 3).

The gBSA caused significant increase (p<0.005, 350%) in the absorbance as compared to nBSA (Fig 3). The AG (5 mM) caused significant decrease (p<0.01, 51%), while PHL treatment (0.5, 1 and 2 mM) caused significant dose dependent decrease [3%, 16% (p<0.05) and 21% (p<0.05)] in the absorbance as compared to gBSA.

### TNBSA assay

**Fig 4:**
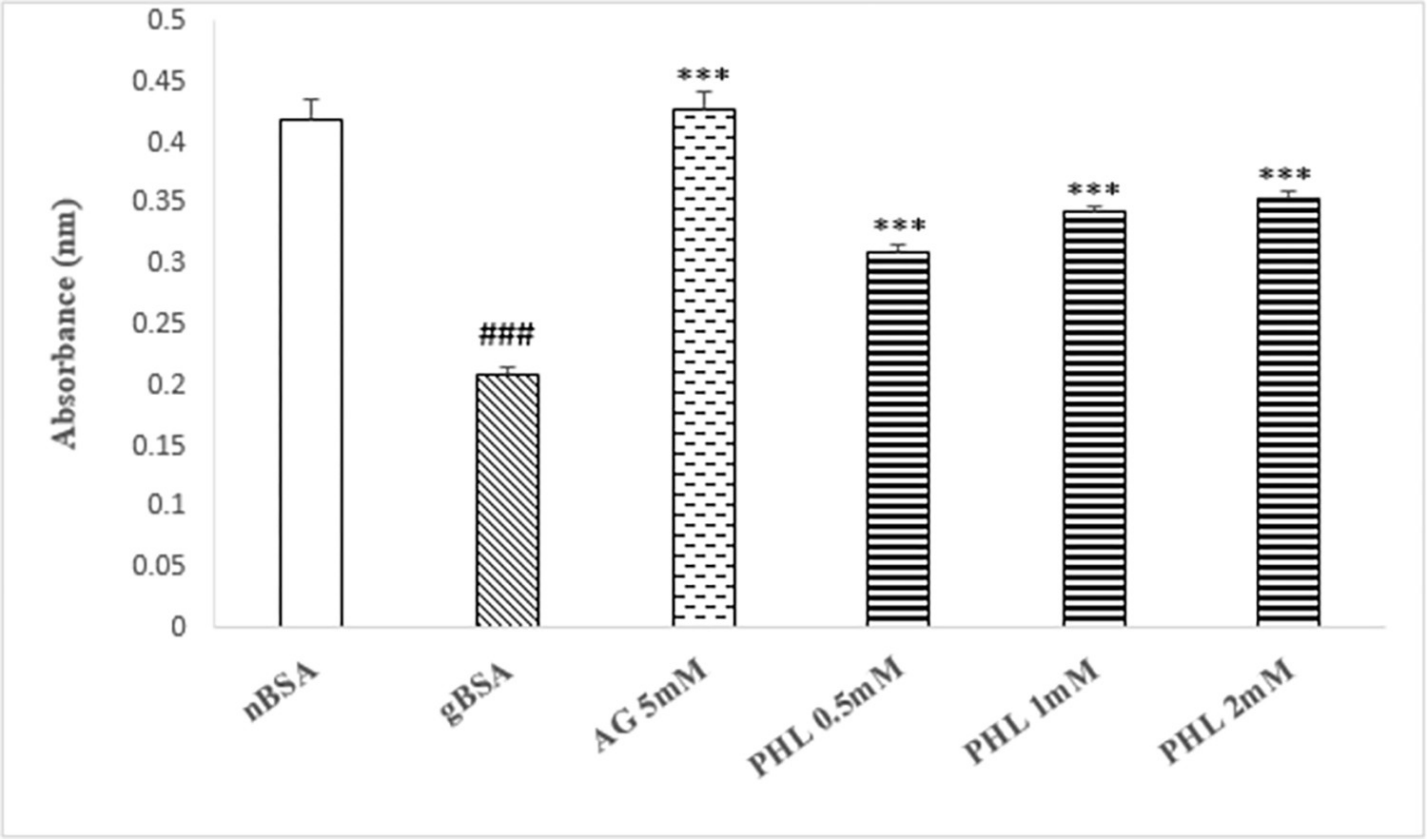
Effect of Phloroglucinol on TNBSA assay. The figure depicts mean ± SEM of absorbance indicative of free lysine in the presence of AG and PHL (n = 3). The gBSA group exhibit reduction in the absorbance as compared to nBSA. The AG and PHL shows significant increase in the absorbance in comparison with gBSA. The hash (###) represents the significant (p<0.005) difference as compared to nBSA while asterisks [* (p<0.05), ** (p<0.01) and ***(p<0.005)] represents the statistical comparison with the gBSA.

The gBSA showed significant decrease (p<0.005, 50%) in the absorbance as compared to nBSA (Fig 4). The AG (5 mM) caused significant increase in absorbance (p<0.005, 100%), while PHL treatment (0.5, 1 and 2 mM) exhibited significant (p<0.005) dose dependent increase (47%, 61% and 67% respectively) in the absorbance as compared to gBSA.

### Thioflavin-T assay

**Fig 5:**
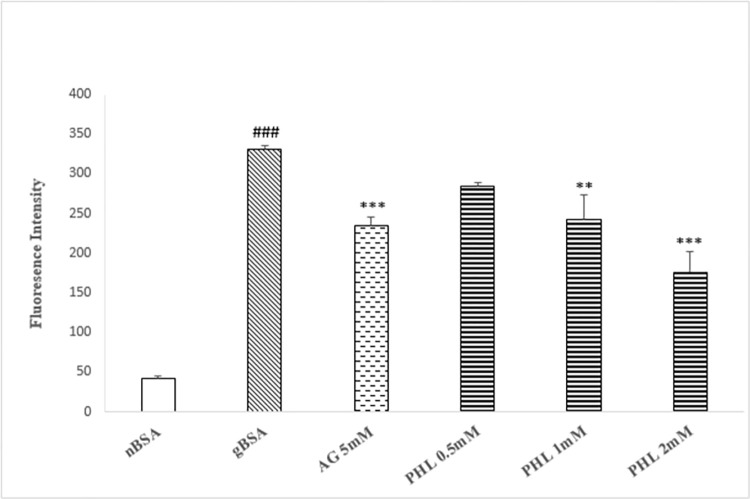
Effect of Phloroglucinol on Thioflavin-T assay. The figure shows the mean ± SEM of ThT fluorescence in the presence of AG and PHL (n = 3). The gBSA group exhibit enhanced fluorescence as compared to nBSA. The AG and PHL treatment groups exhibit significant decrease in fluorescence as compared to gBSA. The hash (###) represents the significant (p<0.005) difference as compared to nBSA while asterisks [* (p<0.05), ** (p<0.01) and ***(p<0.005)] represents the statistical comparison with the gBSA.

The gBSA showed significant increase (p<0.005, 680%) in the fluorescence intensity as compared to nBSA (Fig 5). The AG (5 mM) treatment has significantly (p<0.005) decreased the fluorescence by 29% in comparison with gBSA. The PHL treatment (0.5, 1 and 2 mM) also caused significant dose dependent decrease in the fluorescence intensity [(16%), 37% (p<0.05) and 88% (p<0.005), respectively)] as compared to gBSA.

### Congo Red assay

**Fig 6:**
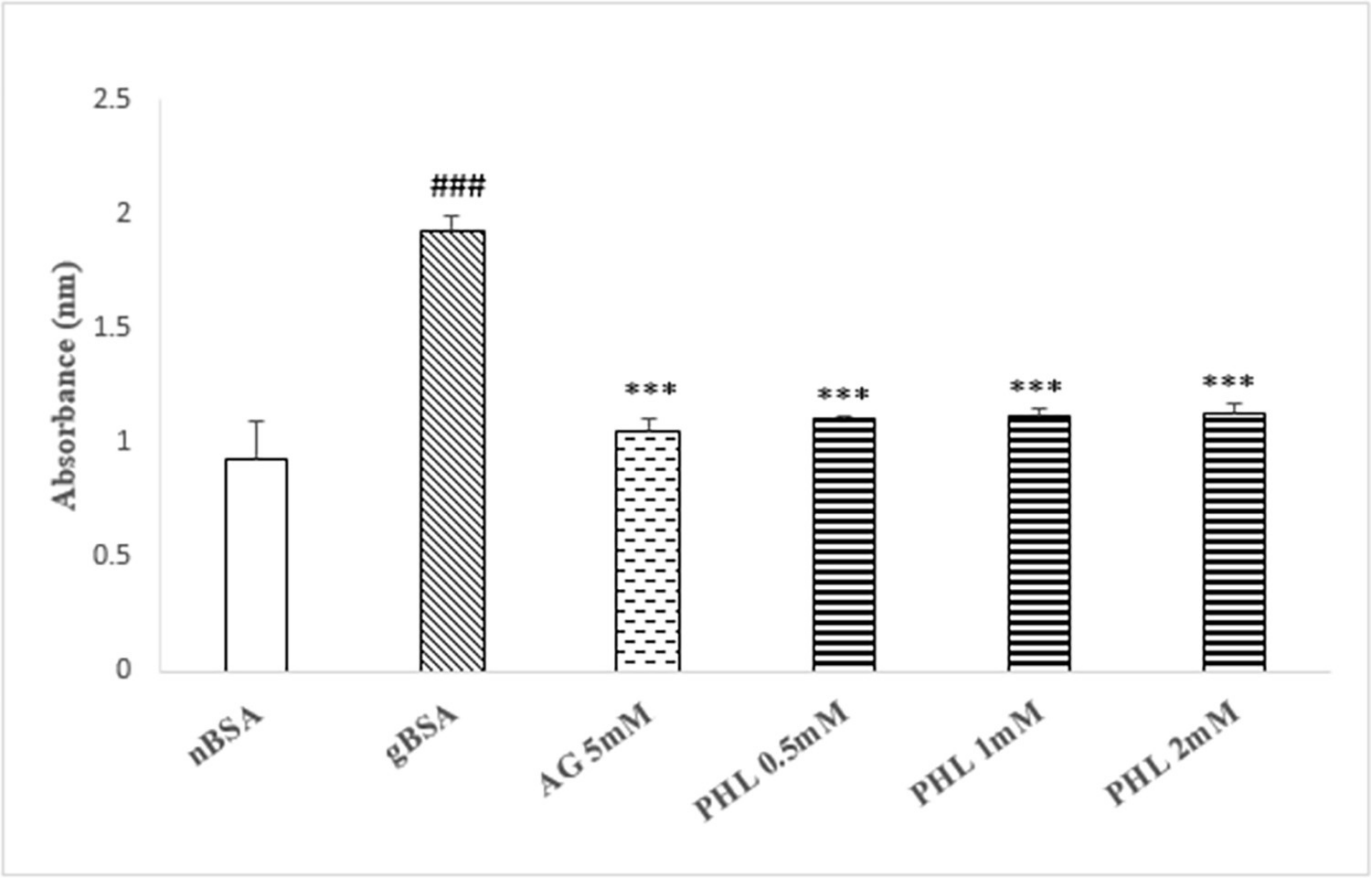
Effect of Phloroglucinol on Congo Red assay. The figure shows the formation of amyloid-like aggregates as absorbance in the presence of AG and PHL. The gBSA group exhibit enhanced absorbance as compared to nBSA. The AG (5mM) and PHL (0.25, 0.5 and 1 mM) demonstrated significant reduction in the absorbance as compared to gBSA. The hash (###) represents the significant (p<0.005) difference as compared to nBSA while asterisks [* (p<0.05), ** (p<0.01) and ***(p<0.005)] represents the statistical comparison with the gBSA. All values are expressed as mean ± SEM of absorbance (n = 3).

The gBSA exhibited significant increase (p<0.005, 105%) in the absorbance as compared to nBSA (Fig 6). The AG (5 mM) treatment has significantly reduced the absorbance (p<0.005, 187%), while PHL treatment (0.5, 1 and 2 mM) caused significant decrease (p<0.005, ~ 42%) in absorbance as compared to gBSA.

### Circular dichroism

**Fig 7:**
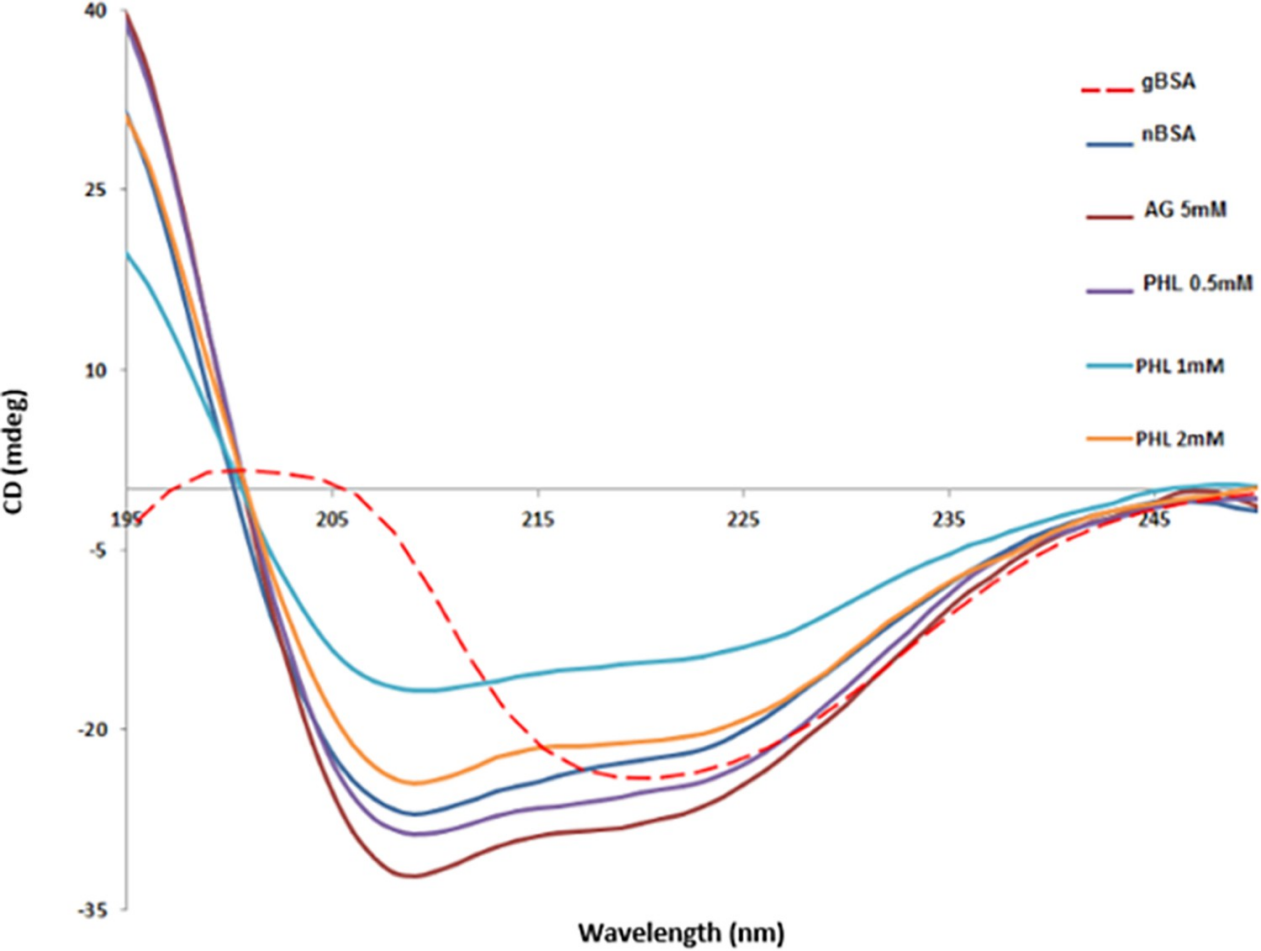
Effect of Phloroglucinol on CD spectra of BSA. The figure shows the CD spectra of BSA underwent glycation reaction with fructose in the presence and absence of AG and PHL. The nBSA showed its characteristic alpha helical spectra with two lambda max at 208 and 222 nm, while the spectra of gBSA exhibited lambda max only at 218 nm, which is indicative of loss of alpha helicity. The AG and PHL exposure gave the BSA spectra similar to that of nBSA, which suggests the preservation of secondary structure of BSA in the presence of fructose.

The nBSA demonstrated the CD spectra with two lambda max at 208 and 222 nm, which is indicative of the alpha helical structure. On the contrary, the spectra of gBSA exhibited lambda max at 218 nm, which advocates the transition of protein structure form alpha helical to beta-pleated sheets (Fig 7). The spectra obtained from AG and PHL treatment groups are similar to that of nBSA, which is suggestive of the preservation of secondary structure of BSA even in the presence of fructose.

### Lysine blockage assay

**Fig 8:**
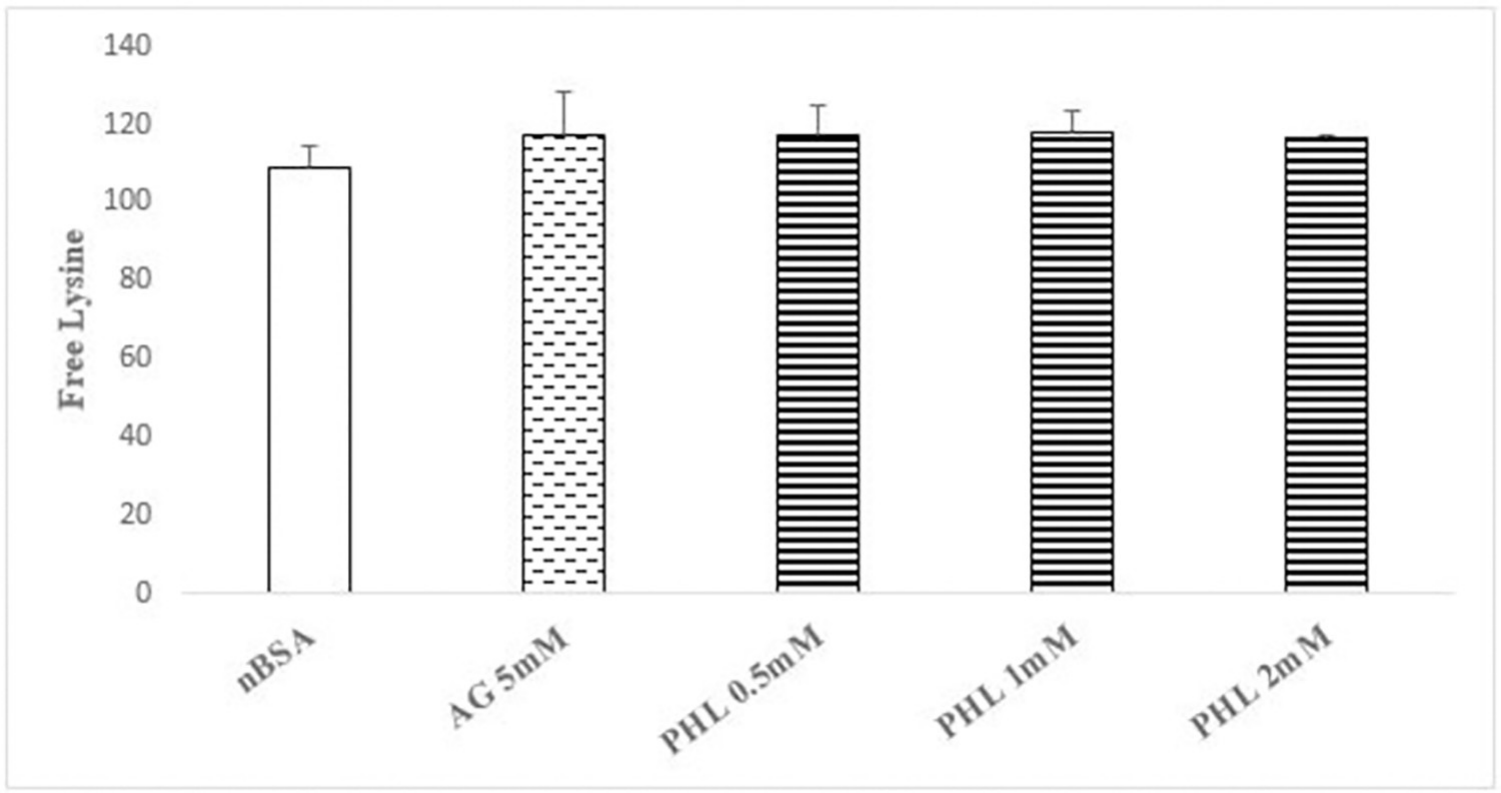
Effect of Phloroglucinol on lysine blockage assay. The figure shows mean ± SEM of fluorescence intensity as lysine blockade in the presence of AG and PHL (n = 3). The data shows the insignificant alterations in fluorescence intensity in both standard as well as test drug.

Our data shows that none of the treatment groups caused significant change in the fluorescence intensity, which advocates that neither AG nor PHL have the ability to block the lysine residues of BSA. Thus, the test agents did not interfere with early fructose-BSA (lysine) interaction to affect the process of glycation (Fig 8).

### Computational study

**Fig 9:**
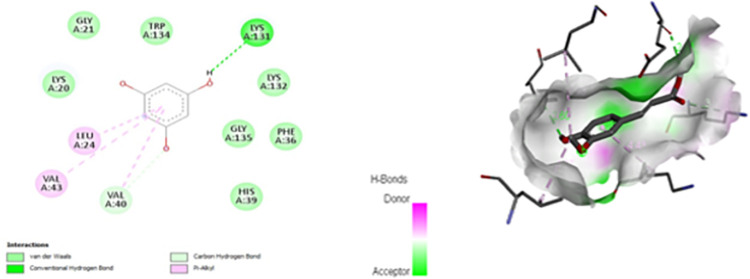
Binding interaction of Phloroglucinol with different residues of BSA. The computational demonstrated interaction of PHL with the GLY, HIS, ILE, LYS, PHE, TRP and VAL residues of BSA.

The molecular docking study showed the interaction of PHL with GLY, HIS, ILE, LYS, PHE, TRP and VAL of BSA (Fig 9). None of these amino acids is catalytic residue i.e. the one involved in mediating the glycation reaction.

### Carbonyl entrapping assay

**Fig 10:**
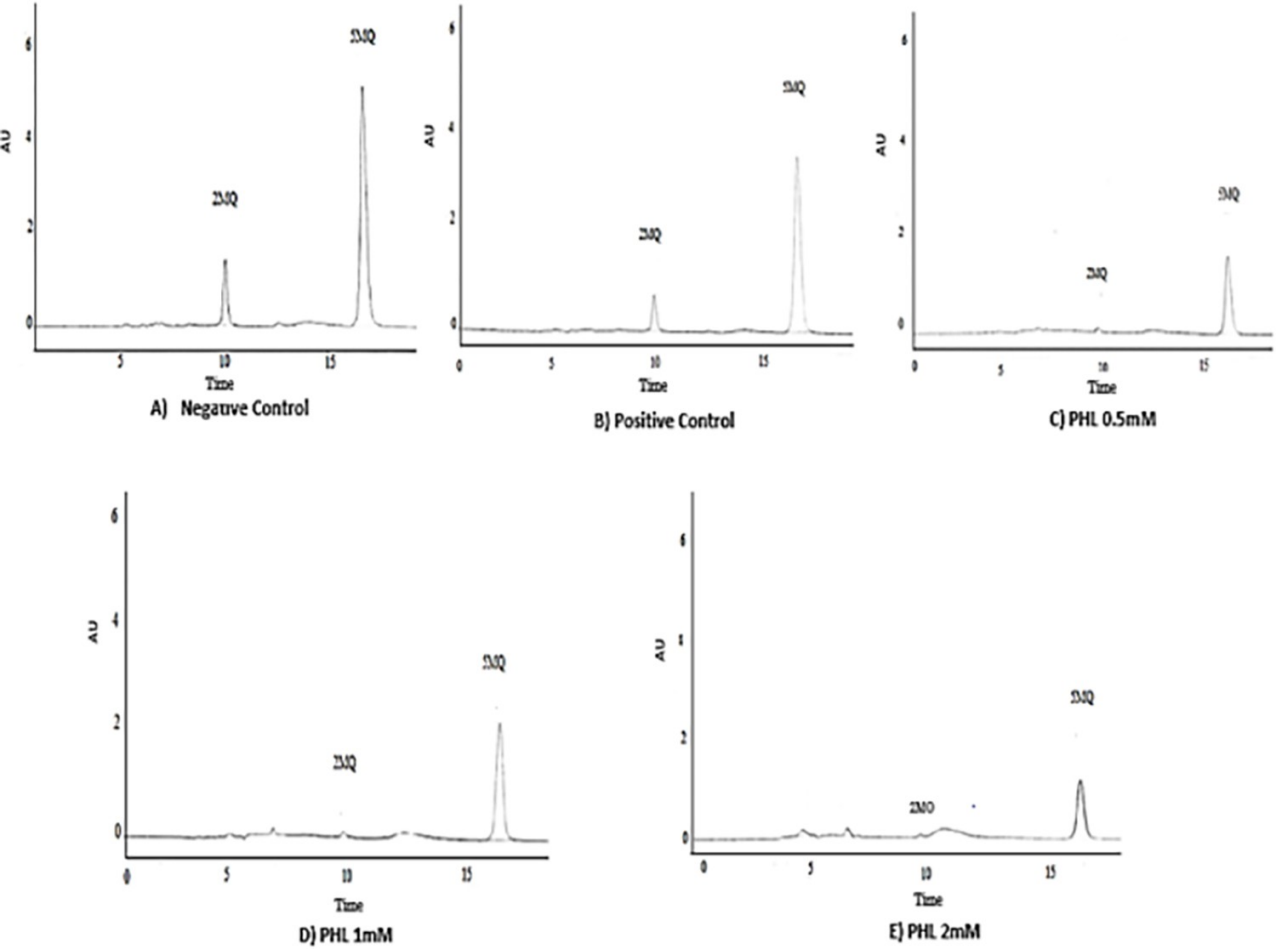
Effect of Phloroglucinol on carbonyl entrapping assay using HPLC. The figure depicts the representative chromatograms of carbonyl entrapping assay in the presence of AG and PHL. The 2-MQ peak was found in the negative control group, while it was significantly reduced following AG and PHL treatments. The percent change following AG treatment was found to 52% whereas in case of PHL, the dose dependent increase in percent inhibitions were [(0.5mM (92%), 1mM (94%), 2mM (97%)] respectively.

The AG (5 mM) caused 52% inhibition in the AUC of 2-MQ. The PHL treatment also caused dose dependent decrease in the AUC [0.5 mM (92%), 1 mM (94%), and 2 mM (97%) respectively]. The representative chromatograms of various treatment groups are shown in Fig 10.

## Discussion

Glycation is among the major underlying mechanisms attributed to ageing and associated morbidities. So far, there is no treatment option available to combat this deleterious phenomenon. Drug repurposing provide the robust and economical means to introduce newer molecule to drug discovery process. Keeping this into account, the present study was designed to evaluate the anti-glycation potential of PHL, an existing anti-spasmodic drug.

The concepts of structure-activity relationship plays a pivotal role in identification of lead molecule for drug discovery program. Search of literature advocates that the abundance of phenolic hydroxyl groups in the benzene ring positively co-relate with the anti-glycation activity [29]. Based on aforesaid chemical attributes, the PHL was selected for present study since it belongs to the polyphenolic class of chemicals and has multiple -OH (hydroxyls) groups in the ring structure [30]. In order to assess the anti-glycation activity, the ability of AGEs to demonstrate intrinsic fluorescence possesses significant utility. Our *in vitro* model involving fructose and BSA exhibited the enhanced fluorescence as compared to the nBSA, which is suggestive of the formation of AGEs (Fig 2). Furthermore, in similarity with standard AG, the PHL (0.5, 1 and 2 mM) also caused significant reduction in the fluorescence as compared to gBSA, which is indicative of its anti-glycation ability. Hence, our preliminary findings support that PHL merits further investigation as anti-glycation molecule.

Glycation is the complex phenomenon, which involve cascade of events such as the formation of early fructose amine adducts, intermediate carbonyl compound and end products at advance stage [31]. Our data regarding fructosamine content exhibited significantly enhanced absorbance in gBSA as compared to nBSA (Fig 3), which is indicative of enhanced glycation reaction. In similarity with AG, the PHL caused dose dependent (0.5, 1 and 2 mM) decrease in the absorbance as compared to gBSA. Hence, the lower fructosamine load in reaction mixture containing PHL further confirms its anti-glycation potential. Search of literature revealed that fructose reacts with lysine in BSA to initiate the glycation phenomenon [32]. Hence, the availability of free lysine provides information regarding the intensity of reaction, which was also assessed in present study using TNBSA assay [33]. Our data shows significant reduction in free lysine in gBSA as compared to nBSA (Fig 4). Moreover, in similarity with AG, PHL treated samples revealed higher availability of free lysine as compared to the gBSA (Fig 5). This suggest that PHL treatment has the ability to interfere in the fructose-lysine interaction thereby inhibiting glycation.

Conformational changes in the protein structure occurs during the process of glycation [34]. Originally, BSA is a larger alpha helical protein of around 66 kDa having 583 amino acid residues [35]. Upon reaction with reducing sugar, BSA transform its structure from alpha-helical to beta pleated sheets [25]. To assess this change, Thioflavin-T and Congo Red tests were used. In conformation with existing literature, the glycated BSA showed the enhance values of fluorescence and absorbance for ThT (Fig 5) and CR (Fig 6), respectively, as compared to nBSA. This is suggestive of formation of amyloid-like aggregates of BSA in these samples. However, these values were found to be significantly reduced in both AG and PHL treated samples. Hence, it can be deduced that both of these drugs has the ability to prevent the glycation induce changes in the secondary structure of BSA. Circular dichroism (CD) is among the most reliable techniques for evaluating the secondary structure of proteins. The native BSA with alpha helical rich structure is reported to give distinct positive band at 192 nm and two negative bands around 208 and 222 nm [36], which was observed in our nBSA samples too (Fig 7). Furthermore, the positive bands around 195 nm and a distinct negative band at 218 nm indicate change in protein conformation from alpha helical to beta-pleated sheets, which was observed in our gBSA samples. It was worth noting that CD spectra of AG and PHL treated samples were found to be similar to that of nBSA, which advocates for their ability to preserve the alpha helicity of BSA in glycation prone environment.

Lysine blockade and Carbonyl trapping presents two important pharmacological targets for anti-glycation compounds [37]. Hence, both of these were assessed to identify the potential mode of action of PHL. Our data for lysine blockade action (OPA) assay revealed lack of any such activity following AG treatment (Fig 8). This is in agreement with the existing literature as AG actually belongs the class of carbonyl entrapper [38]. Hence, this validate our methodology. It is of note that the PHL also did not demonstrate any lysine blocking action at any of the tested dose. The lysine blockade activity was also assessed using computational approach. Search of literature revealed that fructose binds with certain catalytic residues in BSA, especially LYS-524 to initiate the reaction of glycation [25]. Our computational data demonstrate that PHL failed to engage any of the catalytic residue of BSA reported to be pivotal in mediating the glycation reaction (Fig 9). Hence, this further confirms that lack of lysine blockade as potential mechanism of anti-glycation action of PHL.

MGO trapping assay has been used to identify the possible anti-glycation mechanism of test compound [39]. In our study, the AG showed significant reduction in the 2-MQ peak (Fig 10), which is suggestive of the entrapping of MGO thereby preventing its derivatization to 2-MQ. This further validate our experimental settings as AG is well-known carbonyl entrapper [25]. It is of note that significant reduction in the AUC of 2-MQ was also observed in PHL treated samples. This is indicative of MGO trapping as potential mechanism of anti-glycation by PHL. Search of literature revealed that scavengers of reactive oxygen species also possess this ability against reactive carbonyl species thereby inhibiting the phenomenon of glycation [40, 41]. In this regard, PHL has been reported earlier to possess the antioxidant action [42, 43], which further supports its carbonyl entrapping activity observed in present study.

Search of literature revealed that PHL has previously been reported to inhibit the process of glycation [44, 45]. However, these earlier findings are preliminary in nature, which involves measurement of AGEs intrinsic fluorescence, fructosamine load and ThT binding as an evidence of anti-glycation potential. However, the present study provides a more comprehensive information, which highlight the role of specific chemical structure of PHL in exhibiting anti-glycation activity. Additionally, the CD analysis provides the direct evidence of preservation of secondary structure of BSA by PHL in glycation prone environment. Most importantly, to the best of our knowledge, this study sheds light for the first time on the mechanism of anti-glycation action of PHL. Hence, the present study has advanced our understanding regarding the anti-glycation potential of PHL. Having said this, the limitations of present study include utilization of single protein-sugar model in assessing the anti-glycation potential. Furthermore, there also lacks *in vivo* set of experiments involving the use of laboratory animals for demonstrating anti-glycation potential of PHL. Future studies will be aimed at addressing the aforementioned limitations.

## Conclusion

PHL has the ability to inhibit the formation of AGEs, which can be attributed to its ability to entrap reactive carbonyl intermediates. Since PHL is already a clinically used drug, therefore after establishing its efficacy as anti-glycation agent at pre-clinical level, a clinical trial will be performed to check its effectiveness in human subjects. Hence, the present study deduce that PHL presents itself as a promising candidate to be re-purposed as anti-glycation agent.

## Abbreviations

AGEs: Advanced Glycation End Products
PHL: Phloroglucinol
BSA: Bovine Serum Albumin
CD: Circular Dichorism
gBSA: Glycated BSA
nBSA: Native BSA
NBT: Nitroblue Tetrazolium
TNBSA: 2,4,6-Trinitrobenzene Sulfonic Acid
